# Thymic Epidermoid Cyst: Clinical and Imaging Manifestations of This Rare Anterior Mediastinal Mass

**DOI:** 10.1155/2016/5789321

**Published:** 2016-11-24

**Authors:** Jawad M. Qureshi, Brian Pagano, Jeffrey Mueller, Lana Schumacher, Claudia Velosa, Matthew S. Hartman

**Affiliations:** ^1^Department of Radiology, Allegheny Health Network, Pittsburgh, PA, USA; ^2^Temple University School of Medicine, Philadelphia, PA, USA; ^3^Department of Surgery, Allegheny Health Network, Pittsburgh, PA, USA; ^4^Department of Pathology, Allegheny Health Network, Pittsburgh, PA, USA

## Abstract

Thymic epidermoid cysts are an extremely rare entity. These arise from epidermal cells that migrate to the thymus. The radiologic diagnosis of this rare lesion is challenging. We describe a case of an otherwise healthy 35-year-old woman who presented with an acute onset of chest pain and shortness of breath. She was found to have an anterior mediastinal mass. The imaging findings were, however, not characteristic for any single diagnostic entity. Since the imaging was inconclusive, surgical resection was performed for definitive diagnosis. The mass was found to be a thymic epidermoid cyst. This case underlines the significance for radiologists to be aware that epidermoid cysts can occur in the thymus and should be considered in the differential diagnosis for a heterogeneous anterior mediastinal mass.

## 1. Introduction

Thymic epidermoid cysts are extremely rare with only three cases described in literature. These are benign and carry an overall good prognosis. However, the location is atypical and imaging findings are nonspecific. Despite their benign nature, surgical resection is required to exclude malignancy and attain a definitive tissue diagnosis.

## 2. Case History

**Figure 1:**
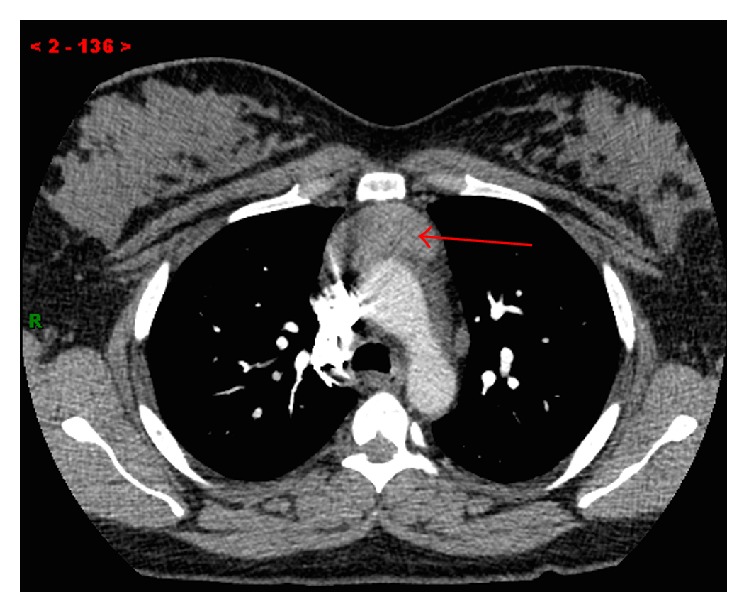
Axial CTA through the level of the aortic arch shows a homogenous anterior mediastinal mass (red arrow).

**Figure 2:**
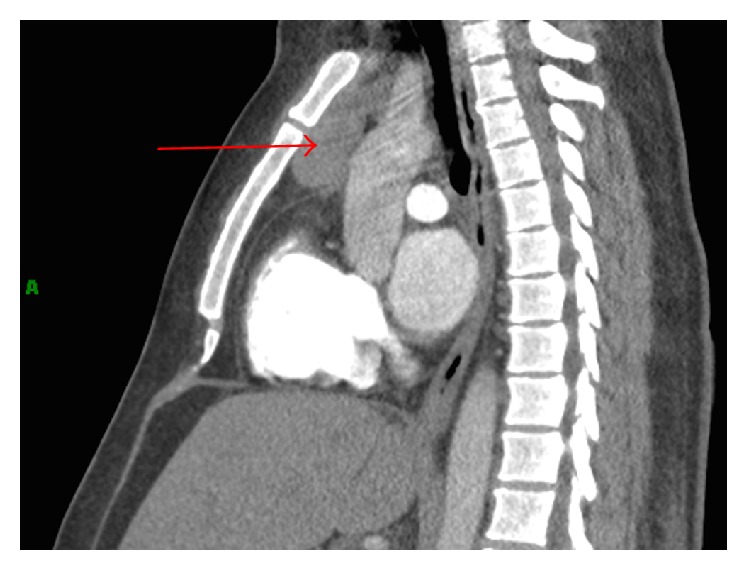
Sagittal reconstruction shows the same mass in the vertical plane (red arrow).

**Figure 3:**
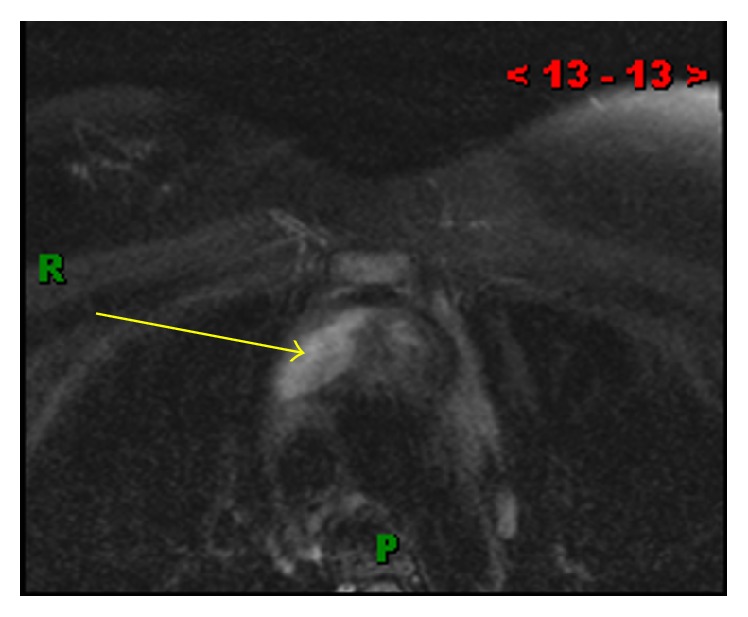
(MR T2) axial T2 fat saturated image showing a hyperintense heterogenous mass suggestive of cystic components (yellow arrow).

**Figure 4:**
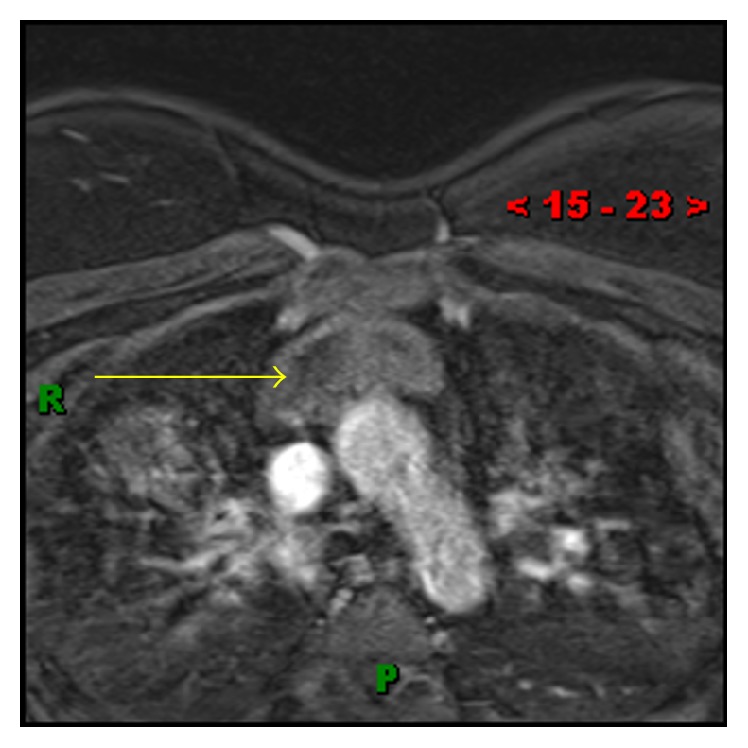
(MR T1 postcontrast) heterogeneous anterior mediastinal mass (yellow arrow) without obvious enhancement.

**Figure 5:**
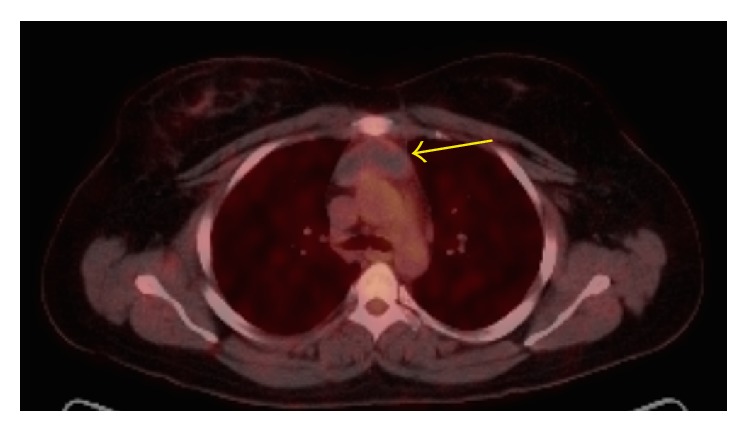
(PET) no significant metabolic activity in the anterior mediastinal mass (yellow arrow).

A 35-year-old lady presented with chest pain and shortness of breath, one week after undergoing a C-section. She was evaluated with a chest computed tomography angiography (CTA) to evaluate possible pulmonary emboli. The chest CTA was negative for pulmonary emboli but incidentally demonstrated a homogenous 5 cm mass in the anterior mediastinum (Figures 1 and 2). The patient was scheduled for a positron emission tomography CT (PET CT) which showed no significant FDG activity in the mass (Figure 5). Follow-up magnetic resonance imaging (MRI) of the chest demonstrated a nonenhancing, heterogeneous anterior mediastinal mass with cystic components and no macroscopic fat (Figures 3 and 4).

**Figure 6:**
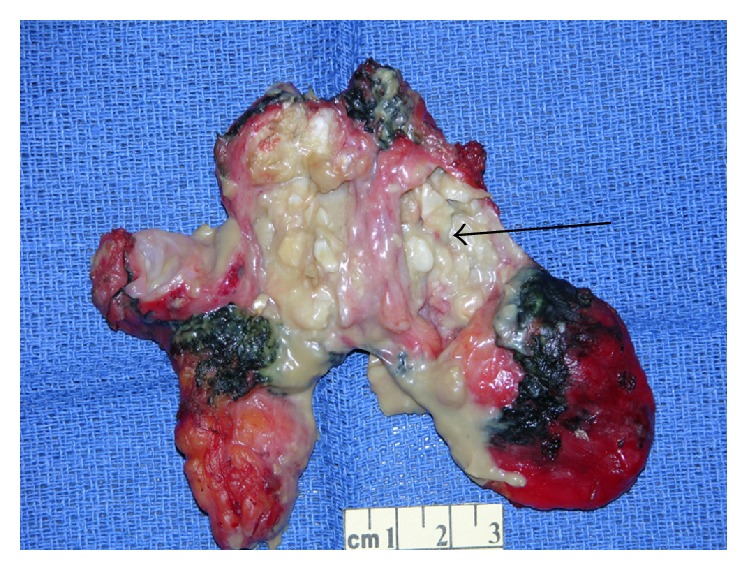
Gross specimen with keratinaceous debris within the cyst (black arrow).

**Figure 7:**
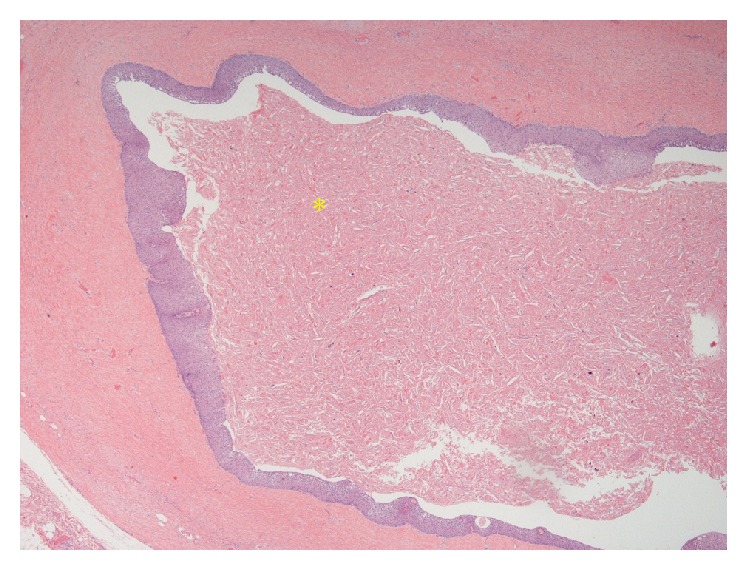
H&E 10x image showing abundant keratin debris (*∗*) within the cyst.

**Figure 8:**
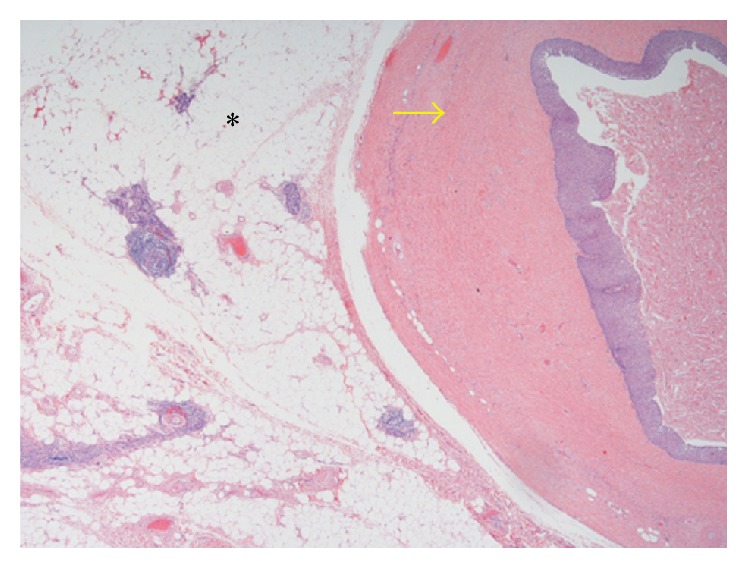
H&E 2.5x image showing the epidermoid cyst wall (yellow arrow) and the normal thymus tissue (*∗*).

CT-guided needle biopsy was performed for definitive diagnosis. This showed benign squamous and fibroconnective tissue and was inconclusive. She subsequently underwent a right robotic assisted thoracoscopy for resection of the mass. A 9.5 cm × 7.0 cm × 3.0 cm soft, round mass with a red, glistening capsule was resected following careful dissection from the adhering mediastinal structures. The surgical specimen was submitted for pathological analysis (Figure 6). The final histopathology of the surgical specimen showed a benign epidermoid cyst, with abundant internal keratin debris, that was attached to benign thymic tissue (Figures 7 and 8). The patient had a good outcome and is currently asymptomatic.

## 3. Discussion

Thymic epidermoid cysts are an extremely rare entity. To the best of our knowledge, this is the 4th reported case. Rare cases of epidermoid cysts have been reported within the spleen, kidney, and the GI and GU tracts [1].

The exact etiology of thymic epidermoid cysts remains unknown. Developmentally, the thymus forms primarily from epithelial cells derived from the endoderm with a mesenchymal thymic remnant. Epidermoid cysts are sequestration cysts that form by proliferation of epidermal cells that arise from the ectoderm within an unusual location within the thymus [2]. Acquired epidermoid cysts in the thymus are hypothesized to result from epidermal tissue migration into the anterior mediastinum and their subsequent proliferation within the thymus. Congenital epidermoid cysts may potentially form in the thymus, as in other locations. However, no confirmed case has been reported in the literature to date [3].

There has been a case report of an acquired, posttraumatic thymic epidermoid cyst. This was thought to result from the introduction of epidermoid cells into the thymus following trauma [4]. Epidermoid tissue may be introduced in the thymus following surgery as well. One case report has suggested an association between Gardner's syndrome and the development of thymic epidermoid cysts [5]. However, our patient did not have gastrointestinal or other abdominal signs or symptoms.

The clinical presentation is variable. In the past, epidermoid cysts in the thymus have been diagnosed in asymptotic patients. They have also been found during the workup for chest pain, dyspnea, fever, or hemoptysis. A history of chest trauma or recent surgery may be present. There was no history of trauma in our patient. She first noticed her symptoms towards the end of pregnancy.

The imaging findings are nonspecific, which may be misleading for the radiologists. The differential diagnosis includes thymic hyperplasia, teratoma, thymic neoplasm, or lymphoma. Due to its rarity, an epidermoid cyst of the thymus is not typically part of the differential diagnosis (Table 1). In our patient, the chest CTA demonstrated nonspecific findings of a homogenous anterior mediastinal mass. The subsequent PET CT showed no significant metabolic activity. In retrospect, in our patient the MRI was the most suggestive imaging test for an epidermoid cyst. The MRI showed a heterogenous mass with cystic components. Typically, thymic hyperplasia has a homogenous mass which may lose signal on out-of-phase MR imaging. Although this mass has cystic components, typical thymic cysts are not heterogeneous. Lymphoma can be heterogenous and necrotic but usually enhances and is metabolically active on PET-CT. Teratomas are commonly heterogenous but usually contain macroscopic fat and/or calcifications. There was no obvious macroscopic fat on either CT or MRI in our patient. Epidermoid tumors can show restricted diffusion in other parts of the body, particularly in the brain. Unfortunately, diffusion weighted imaging was not utilized as it is not part of the usual anterior mediastinal mass protocol. A more definitive diagnosis could not be made based on these nonspecific findings on imaging. Therefore, the patient required surgical resection to reach a diagnosis.

Pathology showed that the cystic mass was attached to the normal thymus. The epithelial lining of the cyst consisted of stratified squamous epithelium with abundant keratin debris present within the cyst. No other epithelial or mesenchymal components were identified. These findings were consistent with a thymic epidermoid cyst.

## 4. Conclusion

Epidermoid cysts can occur anywhere in the body. However, the thymus is a very unusual location. This is only the fourth reported case, to the best of our knowledge. These may be found incidentally on asymptomatic patients or on routine workup for chest pain and shortness of breath. A history of trauma or surgery may be associated. Imaging findings are nonspecific. That being said, surgical resection may eventually be necessary to reach a definitive diagnosis. While extremely rare, this entity should be considered in the radiologic differential diagnostic of a heterogeneous anterior mediastinal mass.

## Figures and Tables

**Table 1:** Differential diagnosis of an anterior mediastinal mass.

	CT	PET/CT	MRI	Enhancement pattern
Thymic epidermoid cyst	Heterogeneous	Not FDG avid	Heterogenous	Possible restricted diffusion on DWI

Thymic hyperplasia	Homogenous, soft tissue attenuation	Mildly FDG avid; difficult to exclude malignancy due to physiologic uptake in the thymus	Homogenous	Loss of signal on out of phase

Teratoma	Heterogenous, may contain fat/calcification	Not FDG avid	Heterogenous, may contain fat and calcification	Heterogenous

Thymic neoplasm	Focal mass; possible metastases, local invasion, and/or lymphadenopathy	Mildly FDG avid; difficult to exclude malignancy due to physiologic uptake in the thymus	Focal mass; possible metastases, local invasion, and/or lymphadenopathy	T1 isointense to muscle/normal thymus; heterogenous on T2WI

Lymphoma	Enlarged, heterogeneously enhancing mass	FDG avid	Heterogenous, could be necrotic	Shows enhancement

## References

[1] Sahoo M. R., Gowda M. S., Behera S. S. (2013). Unusual site and uncommon presentation of epidermoid cyst: a rare case report and review of literature. *BMJ Case Reports*.

[2] Boehm T. (2008). Thymus development and function. *Current Opinion in Immunology*.

[3] Pear B. L. (1970). Epidermoid and dermoid sequestration cysts. *The American Journal of Roentgenology, Radium Therapy, and Nuclear Medicine*.

[4] Monaco F., Barone M., Monaco M. (2015). Intrathymic epidermoid cyst: a very rare condition. *Asian Cardiovascular & Thoracic Annals*.

[5] Delamarre J., Dupas J. L., Muir J. F., Deschepper B., Sevestre H., Capron J. P. (1987). Gardner's syndrome and epidermoid cyst of the thymus. *Gastroenterologie Clinique et Biologique*.

